# Impact of Serum Uric Acid Levels on the Diagnosis of Coronary Vasospastic Angina

**DOI:** 10.3390/jcm14207200

**Published:** 2025-10-13

**Authors:** Nao Tamura, Yuichi Saito, Kazuya Tateishi, Ken Kato, Hideki Kitahara, Yoshio Kobayashi

**Affiliations:** Department of Cardiovascular Medicine, Chiba University Hospital, Chiba 260-0856, Japan; o8050487444@gmail.com (N.T.);

**Keywords:** vasospastic angina, uric acid, acetylcholine, diagnosis

## Abstract

**Background**: Given that insulin resistance and lower levels of high-density lipoprotein cholesterol are reportedly associated with the development of coronary vasospasm, metabolic disorders may play a significant role in the underlying mechanisms of vasospastic angina (VSA). In this context, however, the impact of serum uric acid (SUA) levels on the diagnosis of VSA remains unclear. **Methods**: From May 2012 to March 2025, a total of 947 patients undergoing intracoronary acetylcholine (ACh) provocation tests for diagnosing VSA were included. Positive diagnosis of ACh provocation testing was defined as angiographic coronary spasm accompanied by chest symptoms and/or ischemic electrocardiographic changes. The primary interest of this study was to evaluate the potential relationship between SUA levels and ACh provocation test results. **Results**: Of the 947 patients, 497 (52.5%) had positive ACh provocation testing (i.e., VSA). Patients with positive ACh tests had significantly higher SUA levels than their counterparts (5.4 ± 1.6 vs. 5.2 ± 1.5 mg/dL, *p* = 0.039). The receiver operating characteristics curve analysis showed that SUA levels were predictive of positive ACh test results (area under the curve 0.538, best cut-off value 5.4 mg/dL, *p* = 0.040). In the multivariable logistic regression analysis, male sex and current smoking were identified as predictors of positive ACh testing, while SUA levels were not. **Conclusions**: Patients diagnosed with VSA had a higher SUA level than patients without VSA. However, this association was confounded with other factors, such as male sex and current smoking.

## 1. Introduction

Vasospastic angina (VSA) is one of the major etiologies of ischemic heart disease, characterized by transient myocardial ischemia and chest symptoms, leading to acute myocardial infarction and cardiac death [1,2]. Although the precise underlying mechanisms remain unclear, the pathogenesis of VSA involves endothelial dysfunction and vascular smooth muscle cell hyper-reactivity [3]. Several contributing factors to VSA have been identified, including smoking, genetic polymorphisms in the aldehyde dehydrogenase 2 (ALDH2) gene, and previous coronary stenting [4,5,6]. Given that insulin resistance and lower levels of high-density lipoprotein (HDL) cholesterol are reportedly associated with VSA, metabolic syndrome is believed to play a significant role in the development of coronary spasm [4,7]. In this context, uric acid, the final product of purine metabolism, might be another key factor in the pathogenesis of VSA. Elevated serum uric acid (SUA) levels are often observed in individuals with metabolic syndrome and can promote vascular inflammation and reactive oxygen species generation in coronary endothelial cells [8]. In the purine metabolism, uric acid is mainly regulated by xanthine oxidoreductase (XOR), converting hypoxanthine to xanthine and xanthine to uric acid [9]. During metabolism, XOR generates oxygen species and promotes inflammatory reactions, resulting in endothelial dysfunction, as shown in previous studies [10]. The imbalance between endothelium-derived vasodilating and vasoconstricting factors impairs homeostasis in patients with endothelial dysfunction, which is a major etiology of VSA along with vascular smooth muscle cell hyper-reactivity [2]. A normal endothelium in the coronary vessels can produce nitric oxide (NO), a potent smooth muscle relaxant, while endothelial dysfunction leads to a lack of endogenous NO and vascular constriction. In a clinical setting, this mechanistic pathway has been established in numerous previous studies showing that several endothelium-dependent vasodilators (e.g., acetylcholine [ACh], ergonovine, histamine, and serotonin) can induce coronary vasospasm in patients with VSA [2]. In patients with an elevated SUA level, previous in vivo studies have shown that higher SUA levels correlated with endothelial dysfunction assessed with several methods (e.g., flow-mediated dilation and reactive hyperemia index) [9]. Therefore, we hypothesized that elevated levels of SUA may be a potential key factor in the pathogenesis of VSA and have clinically relevant diagnostic ability. Indeed, a previous study demonstrated that an elevated SUA level was an independent factor for the VSA diagnosis [11], although the data are scarce. Thus, this study aimed to evaluate the impact of SUA levels on the diagnosis of VSA.

## 2. Materials and Methods

### 2.1. Study Population and Design

**Figure 1:**
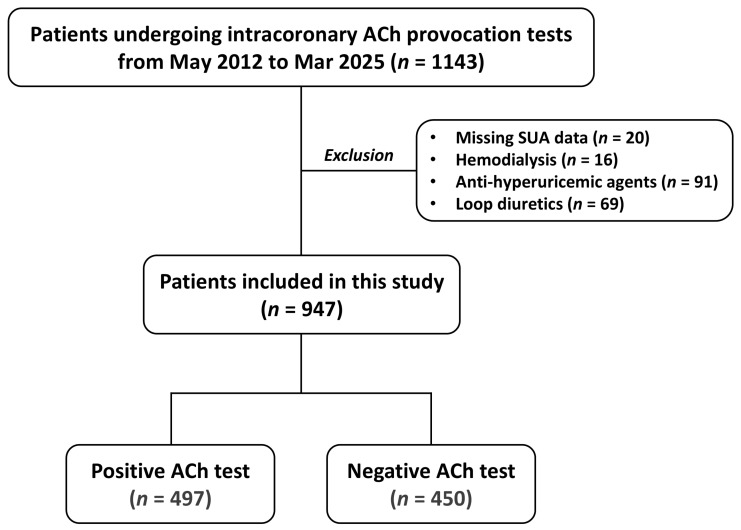
Study flow. ACh—acetylcholine: SUA—serum uric acid.

This single-center study was performed in a retrospective manner at Chiba University Hospital, Japan. From May 2012 to March 2025, a total of 1143 patients underwent intracoronary ACh provocation testing for the diagnosis of VSA. Major exclusion criteria included missing SUA data, maintenance hemodialysis, and medication with anti-hyperuricemic agents and loop diuretics (Figure 1). Thus, a total of 947 patients were included in the current analysis (Figure 1). Cardiovascular risk factors, including hypertension, diabetes, dyslipidemia, and current smoking, were defined based on the Japanese Association of Cardiovascular Intervention and Therapeutics criteria [12]. Hypertension was defined as a prior diagnosis of hypertension or prior antihypertensive treatments, or newly diagnosed hypertension during the hospitalization with systolic blood pressure ≥ 140 mmHg or diastolic blood pressure ≥ 90 mmHg. Diabetes was defined as a prior diagnosis of diabetes or prior glucose-lowering treatments, or glycated hemoglobin ≥ 6.5% during the hospitalization. Dyslipidemia was defined as low-density lipoprotein cholesterol ≥ 140 mg/dL, high-density lipoprotein cholesterol < 40 mg/dL, triglycerides > 150 mg/dL, or a prior diagnosis of dyslipidemia. Low- and high-density lipoprotein cholesterol levels were evaluated in a fasting or non-fasting condition. Other laboratory data, such as hemoglobin and creatinine, were also evaluated. In addition, patients with a history of smoking within one year were defined as current smokers. The triglyceride/high-density lipoprotein cholesterol ratio, a risk marker of cardiometabolic health [13], was calculated with levels of triglyceride divided by high-density lipoprotein cholesterol. This study was conducted in accordance with the Declaration of Helsinki, and the ethics committee of Chiba University Graduate School of Medicine approved the present study. Informed consent was obtained in an opt-out fashion.

### 2.2. Acetylcholine Provocation Test

Intracoronary ACh provocation testing was performed based on the Japanese guidelines [2], as reported previously [4,6]. Briefly, vasodilating agents, such as calcium channel blockers and long-acting nitrates, were discontinued at least 48 h prior to the diagnostic procedures in elective cases, except for short-acting sublingual nitroglycerin if needed. After control coronary angiography, a temporary pacing electrode was placed in the right ventricle. Intracoronary ACh (OvisotⓇ, Daiichi Sankyo, Tokyo, Japan) was then administered in incremental doses of 20, 50, and 100 μg into the left coronary artery and 20 and 50 μg into the right coronary artery, within 20–30 s. One minute after each ACh injection, coronary angiography was performed to evaluate coronary vasospasm. After intracoronary injection of isosorbide dinitrate (1–2 mg), coronary angiography was performed. Angiographic coronary vasospasm was defined as epicardial reduction in coronary vessel diameter ≥ 90% compared with the diameter after the intracoronary injection of isosorbide dinitrate. A positive diagnosis of intracoronary ACh provocation test was defined as significant angiographic coronary spasm accompanied by chest symptoms and/or ischemic electrocardiographic changes [14].

### 2.3. Endpoint and Statistical Analysis

The primary interest of the present study was to evaluate whether patients with an elevated level of SUA had a higher likelihood of positive ACh provocation tests. Patients were categorized into two groups based on the positive or negative ACh test results and the best cut-off value of SUA levels for positive ACh testing. SUA levels were measured before ACh provocation tests.

Statistical analyses were performed using Python (version 3.11.12). Data are presented as mean ± standard deviation or frequency with percentage. Continuous variables were assessed with Student’s *t*-test, and categorical variables were compared using Fisher’s exact test. The receiver operating characteristics (ROC) curve analysis was carried out to evaluate the diagnostic ability of SUA levels for positive ACh tests, with the area under the curve (AUC). The best cut-off value was determined based on Youden’s index. Univariable and multivariable logistic regression analyses were carried out to identify factors associated with positive ACh test results. Variables with a *p*-value < 0.05 in the univariable analysis (age, sex, body mass index, hypertension, diabetes, dyslipidemia, current smoking, prior myocardial infarction, previous percutaneous coronary intervention, SUA, estimated glomerular filtration rate, low-density lipoprotein cholesterol, high-density lipoprotein cholesterol, triglyceride, triglyceride/high-density lipoprotein cholesterol ratio, and glycated hemoglobin) were included in the multivariable model. Sensitivity analysis on sex was also performed. Collinearity among variables was evaluated using the variance inflation factor. When the variance inflation factor was <5, the probability of collinearity was considered low [15]. Given the retrospective design, the formal sample size calculation was not conducted in the present study. A two-sided *p*-value < 0.05 was considered statistically significant.

## 3. Results

A total of 947 patients were included in the present study, of whom 497 (52.5%) had positive intracoronary ACh provocation test results (i.e., VSA). Baseline characteristics are listed in Table 1. Patients with positive ACh tests had significantly higher SUA levels, along with a higher likelihood of male sex and current smokers and lower levels of HDL cholesterol, than those with negative test results (Table 1).

**Figure 2:**
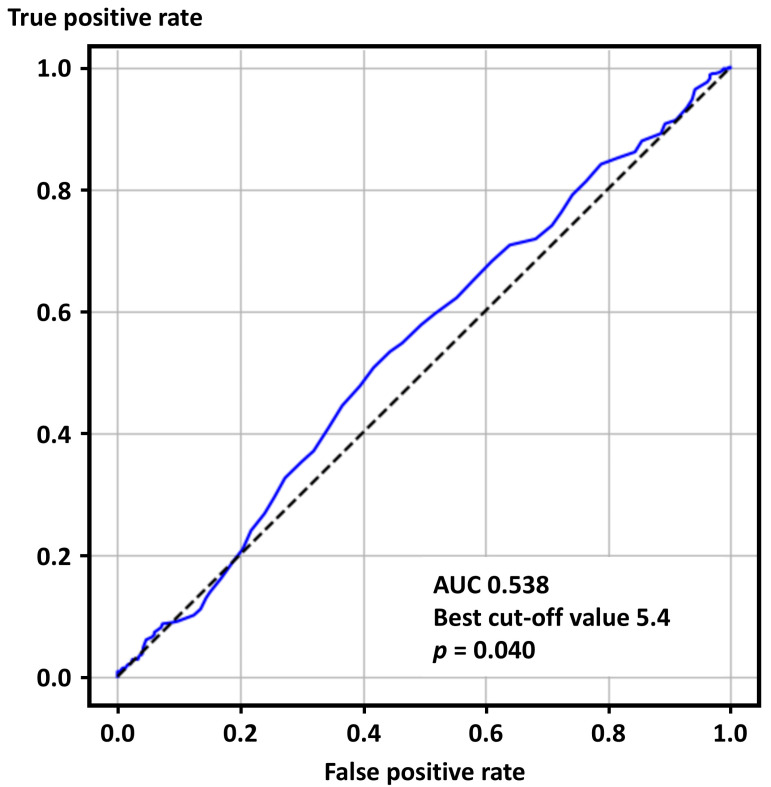
Receiver operating characteristics curve analysis for positive acetylcholine provocation tests. AUC—area under the curve.

The ROC curve analysis showed that SUA levels were predictive of positive ACh tests (AUC 0.538, best cut-off value 5.4 mg/dL, *p* = 0.040) (Figure 2).

When dividing patients into two groups based on the best cut-off value, body mass index and the proportion of male sex and current smokers were higher in those with elevated SUA levels (Table S1).

The probability of positive ACh tests was significantly higher in the group of higher SUA levels than in their counterparts (57.2% vs. 48.3%, *p* = 0.007). Univariable logistic regression analysis indicated that male sex, current smoking, and levels of SUA and HDL cholesterol were factors associated with positive ACh provocation testing, while in the multivariable analysis, male sex and current smoking remained significantly associated with the positive test results (Table 2).

**Figure 3:**
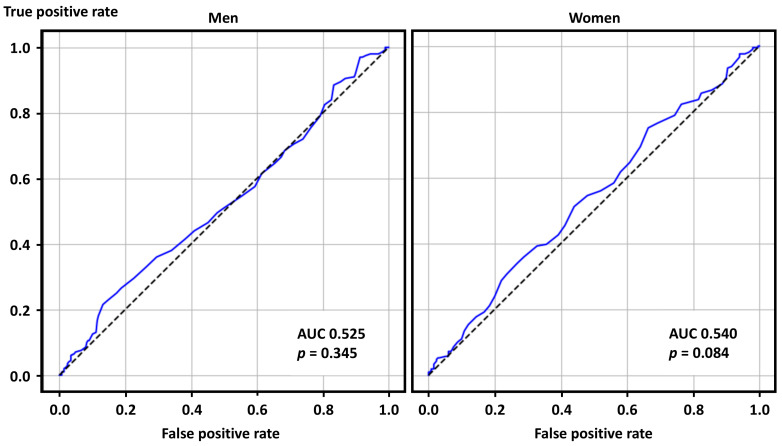
Receiver operating characteristics curve analysis for positive acetylcholine provocation tests in men and women. AUC—area under the curve.

The ROC curve analysis, as a sensitivity analysis, showed that SUA levels were predictive of positive ACh test results in women with a marginal statistical significance but not in men (Figure 3).

## 4. Discussion

The present study demonstrated that among patients undergoing intracoronary ACh tests for the VSA diagnosis, a higher SUA level was associated with positive provocation test results in the univariable analyses. However, the diagnostic ability of SUA levels for VSA was low and was not clinically meaningful. The elevated SUA levels were confounded with several variables, such as male sex and current smoking, resulting in no significant relation between SUA levels and positive ACh test results in the multivariable analysis. Our results suggest that an SUA level lacks independent diagnostic value and should only be considered as an association indicator influenced by confounding factors.

### 4.1. Factors Associated with Coronary Vasospasm

To date, numerous precipitating factors for coronary vasospasm have been reported, including cold stimulation, hyperventilation, ergonovine, and ACh, which are clinically translated into spasm provocation testing [2]. Additionally, various contributing factors to VSA have also been identified, such as smoking, ethnicity (e.g., East Asian), genetic factors (e.g., polymorphisms in the ALDH2), inflammation, myocardial bridging, and previous coronary stenting [4,5,6]. To predict a positive ACh test response, Rinaldi et al. recently proposed the ABCD score, consisting of four items such as acute clinical presentation (positive cardiac troponin), myocardial bridge, high C-reactive protein levels, and dyslipidemia [16]. To improve and simplify the diagnostic scoring system, we developed the modified ABCD score, consisting of acute clinical presentation, myocardial bridge, cigarette smoking, and low HDL cholesterol levels [17]. These findings have contributed to understanding and clarifying the mechanisms of VSA from a clinical perspective. In addition to levels of HDL cholesterol, insulin resistance was reportedly associated with the development of coronary vasospasm. A retrospective single-center study in Korea showed that higher levels of a homeostasis model assessment of insulin resistance were correlated with increased risks of coronary vasospasm induced by intracoronary ACh provocation [18]. Given that insulin resistance and lower levels of HDL cholesterol are important components of metabolic syndrome [19], we hypothesized that a higher SUA level, which is also found in individuals with metabolic syndrome, can be another key factor for VSA. At least in the univariable analysis, some previous reports showed that SUA levels were significantly higher in patients with VSA [5,20]. In a retrospective single-center study in Japan, patients with positive intracoronary ACh tests had a higher SUA level than their counterparts, and the association was significant even in the multivariable analysis [11]. However, these previous studies did not necessarily focus on the impact of SUA levels on the diagnosis of VSA.

### 4.2. Impact of Uric Acid on Coronary Spasm

Uric acid is an end-product of human purine metabolism, mainly regulated by XOR, which converts hypoxanthine to xanthine and uric acid [9]. Despite being recognized as an antioxidant in experimental studies, uric acid can induce oxidative stress and inflammation in endothelial and smooth muscle cells in vessels, leading to endothelial dysfunction [21]. Given that endothelial dysfunction and abnormal NO production are major etiologies of VSA, patients with an elevated SUA level, who are established to have impaired endothelial function in vitro and vivo studies, may be likely to have VSA. Although the causal relationship between hyperuricemia and cardiovascular diseases has been a matter of debate, an elevated SUA level is epidemiologically indicative of the development of hypertension, chronic kidney disease, heart failure, and coronary artery disease, and we have previously reported hyperuricemia as a marker of a broad spectrum of ischemic heart disease [9]. From mechanistic and epidemiological perspectives, it may be reasonable that elevated levels of SUA are associated with VSA. Indeed, previous studies have repeatedly shown that patients with VSA had significantly higher SUA levels than those without, in the three studies reported by Mizuno et al. (5.7 vs. 4.9 mg/dL, *p* = 0.007), Nishino et al. (6.1 vs. 5.5 mg /dL, *p* < 0.001), and Itoh et al. (5.8 vs. 5.2 mg/dL, *p* = 0.006) [5,11,20]. In the multivariable analysis in the three studies, however, the results were conflicting, being marginally significant (*p* = 0.074), significant (*p* = 0.01), and non-significant (*p* = 0.137) [5,11,20]. Importantly, the proportion of men and (current) smokers was numerically or significantly higher in the VSA group than in the non-VSA group in such studies, suggesting a confounding effect with SUA levels. Thus, in the present study, we dedicatedly evaluated the diagnostic impact of SUA levels, excluding patients with maintenance hemodialysis and medication with anti-hyperuricemic agents and loop diuretics. This study ultimately showed that SUA levels were associated with positive ACh test results in the univariable logistic regression analysis (odds ratio 1.09, 95% confidence intervals 1.00–1.19, *p* = 0.040) but very neutral in the multivariable analysis (odds ratio 1.00, 95% confidence intervals 0.91–1.10, *p* = 0.959) when adjusted with male sex and current smoking. The probability of positive ACh provocation tests was reportedly higher in men and current smokers than in their counterparts [4], and male gender was associated with a smoking habit and elevated SUA levels, suggesting that significantly higher SUA levels in patients with positive rather than negative ACh tests may be confounded by gender and smoking habits. These findings highlight that the causality of elevated SUA levels with VSA may be unlikely. Thus, although some reports indicated XOR inhibitors (e.g., allopurinol) as a possible therapeutic option in patients with VSA [22,23], the effect may also be unlikely according to our results. Nonetheless, a previous single-center study in Japan indicated that plasma XOR activity was significantly associated with the incidence of VSA, independent of SUA levels, particularly in women rather than in men [23]. Therefore, beyond SUA levels, an XOR-targeted therapeutic approach might be beneficial.

### 4.3. Study Limitations

The present study had some limitations. This was a single-center retrospective study, and the limited sample size prevented detailed subgroup analyses, particularly in sex differences. To avoid drug interaction on SUA levels, we excluded patients receiving anti-hyperuricemic agents and loop diuretics. This can enhance the internal validity of this study, while the external validity may be limited. Other medications, such as thiazide-type diuretics, aspirin, angiotensin II receptor blockers (e.g., losartan and irbesartan), and SGLT2 inhibitors, can affect the SUA levels, but the detailed data were missing. Because patients on hemodialysis, anti-hyperuricemic agents, and loop diuretics were excluded, the present study findings cannot apply to such patients, and the potential impact of these drugs on VSA is beyond the scope of this study. In addition, data on alcohol habits and polymorphisms in ALDH2, relevant factors in hyperuricemia and VSA, are lacking. In addition, XOR activity was not measured in the present study. In patients undergoing ACh provocation tests in a non-elective setting, vasodilating drugs, such as calcium channel blockers and long-acting nitrates, were not necessarily discontinued at the time of diagnostic procedures. This study included not only patients without epicardial coronary disease but also those with previous myocardial infarction and stents [24,25,26,27]. Although intracoronary ACh provocation testing was performed based on the guideline recommendations in the present study, the protocol varies widely among institutions, regions, and studies, resulting in significant variations in the diagnostic procedure [28]. Given the limited external generalizability of the present study, it should be acknowledged that the role of SUA in the pathophysiology of VSA cannot be ruled out. The multivariable analysis identified male gender and current smoking as factors significantly associated with VSA, while the level of SUA was not. However, unmeasured significant variables were possible. Further studies are still warranted to investigate the potential role of SUA in the mechanism and diagnosis of VSA.

## 5. Conclusions

Patients with invasively assessed VSA had higher levels of SUA than those without. However, the diagnostic ability of SUA levels was low, and a level of SUA was a neutral factor for positive ACh provocation test results in the multivariable analysis, while male sex and current smoking were significant variables. We believe that our results provide mechanistic insights into the relation of SUA levels to coronary vasospasm. Although this study did not find an independent causal relationship between SUA and VSA, the role of the XOR pathway still requires further validation.

## Figures and Tables

**Table 1:** Patient characteristics according to ACh provocation test findings.

Variable	All (*n* = 947)	Positive ACh (*n* = 497)	Negative ACh (*n* = 450)	*p*-Value
Age (years)	63.0 ± 13.1	62.8 ± 12.6	63.2 ± 13.7	0.638
Men	488 (51.5%)	288 (57.9%)	200 (44.4%)	<0.001
Body mass index (kg/m^2^)	23.6 ± 3.8	23.7 ± 3.7	23.4 ± 4.0	0.215
Hypertension	525 (55.4%)	273 (54.9%)	252 (56.0%)	0.791
Diabetes	171 (18.1%)	86 (17.3%)	85 (18.9%)	0.583
Dyslipidemia	624 (65.9%)	320 (64.4%)	304 (67.6%)	0.378
Current smoker	169 (17.8%)	113 (22.7%)	56 (12.4%)	<0.001
Prior myocardial infarction	77 (8.1%)	46 (9.2%)	31 (6.9%)	0.255
Previous PCI	152 (16.1%)	90 (18.1%)	62 (13.8%)	0.085
Serum uric acid (mg/dL)	5.3 ± 1.5	5.4 ± 1.6	5.2 ± 1.5	0.039
eGFR (mL/min/1.73 m^2^)	75.4 ± 18.3	75.5 ± 17.6	75.2 ± 19.1	0.857
LDL cholesterol (mg/dL)	112.5 ± 33.1	111.7 ± 34.0	113.3 ± 32.1	0.473
HDL cholesterol (mg/dL)	61.6 ± 18.5	60.1 ± 17.9	63.4 ± 19.0	0.008
Triglyceride (mg/dL)	137.9 ± 95.8	139.0 ± 102.0	136.6 ± 88.9	0.711
Triglyceride/HDL cholesterol	2.7 ± 3.0	2.8 ± 3.3	2.6 ± 2.6	0.383
Glycated hemoglobin (%)	5.9 ± 0.9	5.9 ± 0.8	5.9 ± 0.9	0.986
Medical treatment				
Calcium channel blocker	397 (41.9%)	208 (41.9%)	189 (42.0%)	1.000
Long-acting nitrate	145 (15.3%)	95 (19.1%)	50 (11.1%)	<0.001
Antiplatelet	271 (28.6%)	164 (33.0%)	107 (23.8%)	0.002
Statin	356 (37.6%)	189 (38.0%)	167 (37.1%)	0.823
ACE-I or ARB	272 (28.7%)	151 (30.4%)	121 (26.9%)	0.265
β-blocker	141 (14.9%)	74 (14.9%)	67 (14.9%)	1.000
ACh provocation test findings				
Elective tests	727 (76.8%)	375 (75.5%)	352 (78.2%)	0.318
Number of spasm vessels	0.98 ± 0.98	1.69 ± 0.73	0.19 ± 0.50	<0.001
Multivessel spasm	291 (30.7%)	273 (54.9%)	18 (4.0%)	<0.001
Signs of ischemia				
Chest symptoms	549 (58.0%)	448 (90.1%)	101 (22.4%)	<0.001
ECG changes	443 (46.8%)	384 (77.3%)	59 (13.1%)	<0.001
ST-segment elevation	144 (15.2%)	140 (28.2%)	4 (0.9%)	<0.001

ACE-I—angiotensin converting enzyme inhibitor; ACh—acetylcholine; ARB—angiotensin II receptor blocker; ECG—electrocardiogram; eGFR—estimated glomerular filtration rate; HDL—high-density lipoprotein; LDL—low-density lipoprotein; PCI—percutaneous coronary intervention.

**Table 2:** Univariable and multivariable logistic regression analysis for positive ACh tests.

Variable	Univariable		Multivariable	
	OR (95% CI)	*p*-Value	OR (95% CI)	*p*-Value
Age (years)	1.00 (0.99–1.01)	0.638		
Men	1.72 (1.33–2.23)	<0.001	1.48 (1.10–1.99)	0.009
Body mass index (kg/m^2^)	1.02 (0.99–1.06)	0.215		
Hypertension	0.96 (0.74–1.24)	0.741		
Diabetes	0.90 (0.65–1.25)	0.527		
Dyslipidemia	0.87 (0.66–1.14)	0.304		
Current smoker	2.08 (1.47–2.95)	<0.001	1.84 (1.27–2.68)	0.001
Prior myocardial infarction	1.35 (0.84–2.18)	0.210		
Previous PCI (%)	1.38 (0.97–1.97)	0.068		
Serum uric acid (mg/dL)	1.09 (1.00–1.19)	0.040	1.00 (0.91–1.10)	0.959
eGFR (mL/min/1.73 m^2^)	1.00 (0.99–1.01)	0.857		
LDL cholesterol (mg/dL)	1.00 (0.99–1.00)	0.473		
HDL cholesterol (mg/dL)	0.99 (0.98–1.00)	0.009	0.99 (0.99–1.00)	0.143
Triglyceride (mg/dL)	1.00 (1.00–1.00)	0.711		
Triglyceride/HDL cholesterol	1.02 (0.97–1.07)	0.385		
Glycated hemoglobin (%)	1.00 (0.86–1.17)	0.986		

ACh—acetylcholine; CI—confidence interval; eGFR—estimated glomerular filtration rate; HDL—high-density lipoprotein; LDL—low-density lipoprotein; OR—odds ratio; PCI—percutaneous coronary intervention.

## Data Availability

The data that support the findings of this study are available from the corresponding author upon reasonable request.

## Supplementary Materials

The following supporting information can be downloaded at: https://www.mdpi.com/article/10.3390/jcm14207200/s1, Table S1: Patient characteristics according to ACh provocation test findings.

## Abbreviations

The following abbreviations are used in this manuscript:

ACh	Acetylcholine
ALDH2	Aldehyde dehydrogenase 2
AUC	Area under the curve
NO	Nitric oxide
HDL	High-density lipoprotein
ROC	Receiver operating characteristics
SUA	Serum uric acid
VSA	Vasospastic angina
XOR	Xanthine oxidoreductase
