# Supplementary material for: Impact of Serum Uric Acid Levels on the Diagnosis of Coronary Vasospastic Angina
Source: J Clin Med. 2025 Oct 13;14(20):7200. doi: 10.3390/jcm14207200 (PMC12565358; doi:10.3390/jcm14207200)
Supplement: Supplementary file 1 [file jcm-14-07200-s001.zip › Supplemental material.pdf]

Table S1. Patient characteristics according to serum uric acid levels

Variable	High SUA (n = 440)	Low SUA (n = 507)	p-Value
Age (years)	61.5 ± 13.2	64.4 ± 12.9	<0.001
Men	317 (72.0%)	171 (33.7%)	<0.001
Body mass index (kg/m^2)	24.5 ± 4.0	22.8 ± 3.5	<0.001
Hypertension	269 (61.1%)	256 (50.5%)	0.001
Diabetes	92 (20.9%)	79 (15.6%)	0.041
Dyslipidemia	305 (69.3%)	319 (62.9%)	0.045
Current smoker	111 (25.2%)	58 (11.4%)	<0.001
Prior myocardial infarction	43 (9.7%)	34 (6.7%)	0.109
SUA (mg/dL)	6.5 ± 1.2	4.2 ± 0.8	<0.001
eGFR (ml/min/1.73 m^2)	72.8 ± 18.5	77.7 ± 17.7	<0.001
LDL cholesterol (mg/dL)	111.3 ± 34.4	113.5 ± 31.9	0.325
HDL cholesterol (mg/dL)	58.7 ± 18.3	64.3 ± 18.3	<0.001
Triglyceride (mg/dL)	152.5 ± 109.0	124.7 ± 80.2	<0.001
Glycated hemoglobin (%)	5.9 ± 0.8	5.9 ± 0.9	0.886
Previous PCI	73 (16.6%)	79 (15.6%)	0.739
Medical treatment			
Calcium channel blocker	196 (44.5%)	201 (39.6%)	0.145
Long-acting nitrate	29 (6.6%)	39 (7.7%)	0.597
Antiplatelet	133 (30.2%)	138 (27.2%)	0.342
Statin	178 (40.5%)	178 (35.1%)	0.104
ACE-I or ARB	143 (32.5%)	129 (25.4%)	0.020
β-blocker	74 (16.8%)	67 (13.2%)	0.144
ACh provocation test findings			
Number of spasm vessels	1.09 ± 1.01	0.88 ± 0.95	<0.001
Multivessel spasm	152 (34.5%)	139 (27.4%)	0.021
Signs of ischemia			
Chest symptoms	273 (62.0%)	276 (54.4%)	0.022
ECG changes	223 (50.7%)	220 (43.4%)	0.030
ST-segment elevation	73 (16.6%)	71 (14.0%)	0.303
Positive test results	252 (57.2%)	245 (48.3%)	0.007

Patients were divided into two groups according to the best cut-off value of SUA levels for positive ACh provocation tests (i.e. 5.4 mg/dL).

ACE-I—angiotensin converting enzyme inhibitor, ACh—acetylcholine; ARB—angiotensin II receptor blocker, ECG—electrocardiogram, eGFR—estimated glomerular filtration rate, HDL—high-density lipoprotein, LDL—low-density lipoprotein, PCI—percutaneous coronary intervention, SUA—serum uric acid.
